# Supplementary material for: Persistent Organic Pollutants and Fatty Acid Profile in a Typical Cheese from Extensive Farms: First Assessment of Human Exposure by Dietary Intake
Source: Animals (Basel). 2022 Dec 9;12(24):3476. doi: 10.3390/ani12243476 (PMC9774984; doi:10.3390/ani12243476)
Supplement: Supplementary file 1 [file animals-12-03476-s001.zip › animals-2008064-supplementary.pdf]

Table S1. Value of PAH congeners concentration in fresh cheeses.

PAH congeners			Naphtalene	Acenaphtylene	Acenaphtene	Fluorene	Phenantrene	Antracene	Fluoranthene	Pyrene	Benzo(a)Anthracene	Chrysene	Benzo(b)Fluoranthene	Benzo(k)Fluoranthene	Benzo(a)Pyrene	Indeno(123cd)Pyrene	Dibenzo(ah)Anthracene	Benzo(ghi)Perylene	ΣPAH	LMW	HMW	LMW/HMW
LOD (ng/g)			0.01	0.01	0.01	0.01	0.01	0.01	0.01	0.01	0.01	0.01	0.01	0.01	0.01	0.01	0.01	0.01				
TEF	Farm	Season	0.001		0.001	0.001	0.001	0.010	0.001	0.001	0.100	0.001	0.100	0.010	1.000	0.010	1.000	0.100				
Cheese_1	A_org	Winter	6.62	0.01	1.16	1.23	0.01	0.01	0.01	0.01	0.01	0.01	0.01	0.01	0.01	0.01	0.01	0.01	9.07	9.02	0.05	180.36
Cheese_2	B_org	Winter	0.70	0.15	0.16	0.20	0.36	0.01	0.05	0.02	0.01	0.01	0.01	0.01	0.01	0.01	0.01	0.01	1.67	1.57	0.11	14.53
Cheese_3	C	Winter	30.49	0.01	3.46	3.09	7.32	0.01	0.01	0.01	0.01	0.01	0.01	0.01	0.01	0.01	0.01	0.01	44.43	44.38	0.05	887.56
Cheese_4	D	Winter	28.04	0.01	2.22	2.31	6.57	0.28	0.63	0.76	0.01	0.01	0.01	0.01	0.01	0.01	0.01	0.01	40.85	39.42	1.43	27.51
Cheese_5	E	Winter	7.32	0.01	3.01	5.60	9.92	0.01	0.76	0.53	0.01	0.01	0.01	0.01	0.01	0.01	0.01	0.01	27.21	25.87	1.33	19.38
Cheese_6	F	Winter	1.45	0.01	0.52	6.65	13.01	0.66	1.30	1.36	0.01	0.33	0.01	0.01	0.01	0.01	0.01	0.01	25.32	22.30	3.02	7.38
Cheese_7	A_org	Spring	3.21	0.01	0.24	1.21	3.06	0.01	0.14	0.12	0.01	0.01	0.01	0.01	0.01	0.01	0.01	0.01	8.04	7.74	0.30	26.00
Cheese_8	B_org	Spring	14.16	0.01	0.01	0.85	1.85	0.01	0.18	0.22	0.01	0.01	0.01	0.01	0.01	0.01	0.01	0.01	17.32	16.87	0.45	37.80
Cheese_9	C	Spring	21.72	0.01	0.56	0.84	1.75	0.01	0.24	0.25	0.01	0.01	0.01	0.01	0.01	0.01	0.01	0.01	25.41	24.88	0.53	47.04
Cheese_10	D	Spring	3.36	0.01	0.01	0.27	0.47	0.01	0.06	0.03	0.01	0.01	0.01	0.01	0.01	0.01	0.01	0.01	4.24	4.11	0.13	32.77
Cheese_11	E	Spring	13.92	0.01	0.01	0.39	0.88	0.01	0.08	0.08	0.01	0.01	0.01	0.01	0.01	0.01	0.01	0.01	15.40	15.21	0.20	77.18
Cheese_12	F	Spring	11.61	0.74	0.01	0.73	1.69	0.01	0.19	0.08	0.01	0.01	0.01	0.01	0.01	0.01	0.01	0.01	15.09	14.78	0.32	46.75
Cheese_13	A_org	Summer	8.30	0.01	0.01	0.34	0.56	0.01	0.07	0.06	0.01	0.01	0.01	0.01	0.01	0.01	0.01	0.01	9.38	9.21	0.17	54.80
Cheese_14	B_org	Summer	7.92	0.01	0.01	0.47	0.70	0.01	0.05	0.09	0.01	0.01	0.01	0.01	0.01	0.01	0.01	0.01	9.30	9.11	0.18	49.55
Cheese_15	C	Summer	0.13	0.01	0.01	0.27	1.11	0.01	0.36	0.41	0.03	0.10	0.01	0.01	0.01	0.01	0.01	0.01	2.44	1.53	0.92	1.67
Cheese_16	D	Summer	0.20	0.01	0.01	0.45	1.66	0.01	0.44	0.53	0.04	0.21	0.01	0.01	0.01	0.01	0.01	0.01	3.59	2.33	1.26	1.85
Cheese_17	E	Summer	0.13	0.01	0.01	0.11	0.58	0.01	0.31	0.38	0.03	0.11	0.01	0.01	0.01	0.01	0.01	0.01	1.69	0.83	0.86	0.96
Cheese_18	F	Summer	0.20	0.01	0.01	0.13	0.28	0.01	0.04	0.02	0.01	0.00	0.01	0.01	0.01	0.01	0.01	0.01	0.71	0.62	0.09	6.85
min			0.13	0.01	0.01	0.11	0.01	0.01	0.01	0.01	0.01	0.00	0.01	0.01	0.01	0.01	0.01	0.01	0.71	0.62	0.05	0.96
max			30.49	0.74	3.46	6.65	13.01	0.66	1.30	1.36	0.04	0.33	0.01	0.01	0.01	0.01	0.01	0.01	44.43	44.38	3.02	887.56
Average			8.86	0.05	0.63	1.40	2.88	0.06	0.27	0.28	0.01	0.05	0.01	0.01	0.01	0.01	0.01	0.01	14.51	13.88	0.63	84.44
Standard deviation			9.57	0.18	1.11	1.90	3.76	0.16	0.34	0.35	0.01	0.09	0.00	0.00	0.00	0.00	0.00	0.00	13.30	13.09	0.76	204.71

Table S2. Value of PCB congeners concentration in fresh cheeses.

PCB congeners			PCB77	PCB81	PCB(114+118)	PCB105	PCB123	PCB126	PCB156	PCB157	PCB167	PCB169	PCB189	PCB28	PCB52	PCB101	PCB138	PCB153	PCB180	PCB_TEQ(TCDD)	PCB_ndl	Tot_PCB
LOD (ng/g)			0.10	0.02	0.01	0.01	0.01	0.01	0.01	0.00	0.00	0.01	0.01	0.40	0.01	0.01	0.01	0.01	0.01			
TEF	Farm	Season	1E-04	1E-04	1E-04	1E-04	1E-04	1E-01	5E-04	5E-04	1E-05	1E-02	1E-04									
Cheese_1	A_org	Winter	0.05	0.01	0.01	0.01	0.01	0.01	0.01	0.01	0.01	0.01	0.01	0.20	0.01	0.01	0.28	0.19	0.01	5.63E-04	0.69	0.81
Cheese_2	B_org	Winter	0.05	0.01	0.10	0.01	0.10	0.01	0.01	0.01	0.01	0.01	0.01	0.20	0.43	0.01	0.11	0.01	0.01	5.83E-04	0.76	1.07
Cheese_3	C	Winter	0.05	0.01	1.91	0.01	0.01	0.01	0.01	0.01	0.01	0.01	0.01	1.55	6.83	0.01	0.01	0.01	0.01	7.54E-04	8.40	10.43
Cheese_4	D	Winter	0.05	0.01	1.02	0.01	0.01	0.01	0.01	0.01	0.01	0.01	0.01	0.20	6.28	0.01	0.36	0.58	0.01	6.65E-04	7.42	8.56
Cheese_5	E	Winter	0.05	0.01	5.41	0.01	0.01	0.01	0.01	0.01	0.01	0.01	0.01	3.12	21.14	0.01	2.62	0.01	0.01	1.10E-03	26.90	32.42
Cheese_6	F	Winter	0.05	0.01	2.52	0.01	0.01	0.01	0.01	0.01	0.01	0.01	0.01	5.22	29.37	0.01	1.42	1.46	0.18	8.15E-04	37.65	40.44
Cheese_7	A_org	Spring	0.05	0.01	1.45	0.01	0.01	0.01	0.01	0.01	0.01	0.01	0.01	0.20	0.01	0.01	0.27	0.01	0.01	7.08E-04	0.49	2.06
Cheese_8	B_org	Spring	0.05	0.01	2.36	0.01	0.01	0.01	0.01	0.01	0.01	0.01	0.01	0.20	0.01	0.01	0.01	0.01	0.01	7.98E-04	0.23	2.70
Cheese_9	C	Spring	0.05	0.01	0.01	0.01	0.01	0.01	0.01	0.01	0.01	0.01	0.01	0.20	2.25	0.01	0.47	0.01	0.01	5.63E-04	2.94	3.06
Cheese_10	D	Spring	0.05	0.01	0.01	0.01	0.01	0.01	0.01	0.01	0.01	0.01	0.01	0.20	0.18	0.01	0.01	0.01	0.01	5.63E-04	0.40	0.52
Cheese_11	E	Spring	0.05	0.01	0.01	0.01	0.01	0.01	0.01	0.01	0.01	0.01	0.01	0.20	0.50	0.01	0.22	0.01	0.01	5.63E-04	0.94	1.06
Cheese_12	F	Spring	0.05	0.01	0.31	0.01	0.01	0.01	0.01	0.01	0.01	0.01	0.01	0.20	0.81	0.01	0.18	0.18	0.01	5.94E-04	1.38	1.81
Cheese_13	A_org	Summer	0.05	0.01	3.00	0.01	0.01	0.01	0.01	0.01	0.01	0.01	0.01	0.20	0.47	0.01	0.28	0.01	0.01	8.63E-04	0.96	4.08
Cheese_14	B_org	Summer	0.05	0.01	0.53	0.01	0.57	0.01	0.01	0.01	0.01	0.01	0.01	0.20	0.64	0.25	0.17	0.41	0.01	6.73E-04	1.68	2.89
Cheese_15	C	Summer	0.05	0.01	0.10	0.01	0.12	0.01	0.01	0.01	0.01	0.01	0.01	0.20	0.11	0.01	0.19	0.01	0.01	5.84E-04	0.52	0.85
Cheese_16	D	Summer	0.05	0.01	0.18	0.01	0.01	0.01	0.01	0.01	0.01	0.01	0.01	0.20	0.13	0.01	0.20	0.01	0.01	5.80E-04	0.54	0.84
Cheese_17	E	Summer	0.05	0.01	0.11	0.01	0.12	0.01	0.01	0.01	0.01	0.01	0.01	0.20	0.15	0.01	0.16	0.01	0.01	5.85E-04	0.52	0.87
Cheese_18	F	Summer	0.05	0.01	0.01	0.01	0.01	0.01	0.01	0.01	0.01	0.01	0.01	0.20	0.01	0.01	0.01	0.01	0.01	5.63E-04	0.23	0.35
min			0.05	0.01	0.01	0.01	0.01	0.01	0.01	0.01	0.01	0.01	0.01	0.20	0.01	0.01	0.01	0.01	0.01	0.00	0.23	0.35
max			0.05	0.01	5.41	0.01	0.57	0.01	0.01	0.01	0.01	0.01	0.01	5.22	29.37	0.25	2.62	1.46	0.18	0.00	37.65	40.44
average			0.05	0.01	1.06	0.01	0.05	0.01	0.01	0.01	0.01	0.01	0.01	0.72	3.85	0.02	0.39	0.16	0.01	0.00	5.15	6.38
Standard deviation			0.00	0.00	1.48	0.00	0.14	0.00	0.00	0.00	0.00	0.00	0.00	1.34	8.17	0.06	0.64	0.36	0.04	0.00	10.31	11.35

Table S3. Values of contaminants concentration occurrence in fresh cheese samples. ΣPAH*, ΣPCB*, ∑ndl-PCB*, PCB_TEQ* values are normalised at fat gram

Sample ID	Farm	Season*	∑PAH (ng/g)	∑PCB (ng/g)	B(a)Py_TEQ (ng/g)	∑ndl-PCB (ng/g)	PCB_TEQ (ng/g)	∑PAH* (ng/g fat)	∑PCB* (ng/g fat)	∑ndl-PCB* (ng/g fat)	PCB_TEQ* (ng/g fat)
Cheese_1	A_org	Winter	5.34	0.48	1.22E-02	4.11E-01	3.32E-04	21.31	1.90	1.62	1.32E-03
Cheese_2	B_org	Winter	1.02	0.65	8.01E-03	4.68E-01	3.55E-04	4.01	2.57	1.81	1.39E-03
Cheese_3	C	Winter	27.72	6.51	3.50E-02	5.25E+00	4.70E-04	105.81	24.84	20.01	1.80E-03
Cheese_4	D	Winter	24.70	5.18	3.32E-02	4.49E+00	4.02E-04	90.59	18.98	16.46	1.47E-03
Cheese_5	E	Winter	16.75	19.96	2.39E-02	1.66E+01	6.79E-04	58.97	70.27	58.30	2.39E-03
Cheese_6	F	Winter	15.64	24.97	2.65E-02	2.34E+01	5.03E-04	61.73	98.60	91.81	1.99E-03
Cheese_7	A_org	Spring	5.02	1.29	1.23E-02	3.13E-01	4.42E-04	17.78	4.55	1.09	1.57E-03
Cheese_8	B_org	Spring	11.06	1.72	1.85E-02	1.50E-01	5.10E-04	41.16	6.41	0.53	1.90E-03
Cheese_9	C	Spring	15.01	1.81	2.19E-02	1.74E+00	3.33E-04	72.49	8.73	8.39	1.61E-03
Cheese_10	D	Spring	2.58	0.32	9.63E-03	2.50E-01	3.42E-04	9.79	1.20	0.93	1.30E-03
Cheese_11	E	Spring	9.77	0.67	1.71E-02	6.03E-01	3.57E-04	38.68	2.67	2.36	1.41E-03
Cheese_12	F	Spring	10.35	1.24	1.78E-02	9.55E-01	4.07E-04	37.57	4.51	3.45	1.48E-03
Cheese_13	A_org	Summer	5.64	2.45	1.26E-02	5.85E-01	5.19E-04	21.68	9.43	2.23	1.99E-03
Cheese_14	B_org	Summer	6.00	1.87	1.35E-02	1.09E+00	4.34E-04	20.29	6.31	3.66	1.47E-03
Cheese_15	C	Summer	1.53	0.53	1.01E-02	3.31E-01	3.66E-04	5.63	1.95	1.19	1.34E-03
Cheese_16	D	Summer	2.36	0.55	1.24E-02	3.65E-01	3.82E-04	8.23	1.92	1.25	1.33E-03
Cheese_17	E	Summer	1.08	0.55	1.01E-02	3.40E-01	3.74E-04	3.76	1.93	1.16	1.30E-03
Cheese_18	F	Summer	0.47	0.23	8.09E-03	1.54E-01	3.70E-04	1.76	0.85	0.56	1.39E-03
min			0.47	0.23	8.01E-03	1.50E-01	3.32E-04	1.76	0.85	0.53	1.30E-03
max			27.72	24.97	3.50E-02	2.34E+01	6.79E-04	105.81	98.60	91.81	2.39E-03
Average			9.00	3.94	1.68E-02	3.19E+00	4.21E-04	34.51	14.87	12.04	1.58E-03
Standard deviation			8.17	7.00	8.25E-03	6.38E+00	8.89E-05	31.61	26.54	24.26	3.08E-04
